# Traumatic bifid tongue: A rare presentation in a child. Case report

**DOI:** 10.1016/j.amsu.2020.06.040

**Published:** 2020-07-11

**Authors:** Ajaipal S. Kang, Kevin S. Kang

**Affiliations:** a Department of Surgery and Chief of Plastic Surgery, UPMC Hamot, Erie, PA, 16507, USA; b Geisel Dartmouth Medical School, Hanover, NH, 03755, USA

**Keywords:** Tongue trauma, Tongue laceration, Bifid tongue, Tongue lesion, Case report

## Abstract

**Introduction:**

Although most tongue lacerations in children can be treated conservatively, accepted indications for suture repair include complex injury, large flaps, and active bleeding. The purpose of this article is to highlight repair of a unique, severe injury pattern in a child.

**Presentation and treatment:**

A 3-year-old boy fell on a cemented floor causing a midline full-thickness laceration through the median fibrous septum, resulting in a bifid tongue. Given the midline location, neurovascular supply was protected, and following surgical repair, the patient enjoyed an uneventful recovery.

**Conclusion:**

Our case outlines a surgical approach for this unique case of acquired traumatic pediatric bifid tongue, which to our knowledge, has never been reported in the English literature.

## Highlights

• This presented case highlights a unique severe injury pattern in a child.
• A 3-year-old presented with a unique acquired traumatic bifid tongue injury.
• He underwent successful functional and aesthetic surgical repair.
• To the best of our knowledge, this rare presentation of traumatic bifid tongue in a child has not been reported in the English literature.
• The case has been presented based on SCARE guidelines.

## 1 Introduction

The tongue is a complex muscular organ essential for speech, taste, chewing and swallowing [1]. Tongue injuries range in severity from minor lacerations to complete amputation. The most common injury location is the anterior dorsum often as a result of falls, seizures, encephalitis, schizophrenia, self-mutilation, sports injuries, electroconvulsive therapy, or child abuse [[1], [2], [3], [4]]. Young children (age 3–4 years) are particularly prone to such injuries after falls [3,4]. The majority of such injuries are self-limited, without bleeding, non-gaping, and horizontal in orientation [4]. In general, small lacerations of the tongue can be allowed to self-heal when wound margins are in good approximation [5]. Because suturing may predispose the tongue to invasive, closed-space infection and requires general anesthesia for children, a surgical closure is recommended only in cases of larger wounds, profuse bleeding, muscle involvement, or full-thickness injury [[3], [4], [5]].

This case describes the presentation and repair of a rare full-thickness longitudinal tongue laceration in a 3-year-old male. To the best of our knowledge, there are no other reports of a traumatic acquired bifid tongue in a child in the English literature. This case has been reported in line with the SCARE criteria [6].

## 2 Presentation of case

**Fig. 1:**
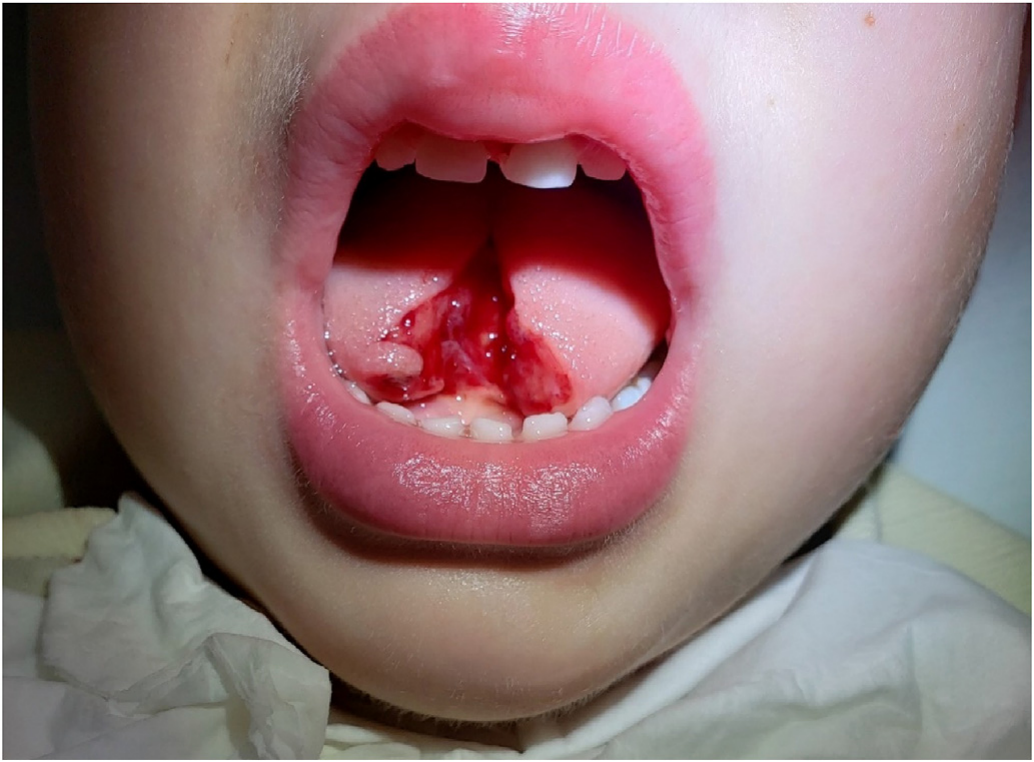
Acquired Bifid Tongue secondary to trauma.

A 3-year-old healthy Caucasian male presented to the Emergency Department by a private car with a laceration of the tongue following a fall-that occurred just prior to arrival. His mother reported that the child was playing in their cemented basement when she heard a loud sound. She did not believe that he landed on any object but found a large amount of blood at the scene. She denied any loss of consciousness, seizures, or neck stiffness. Past medical history, past surgical history, and family history was unremarkable. The patient was not taking any home medications. The child was unable to speak but was without any respiratory distress or hemodynamic compromise. Of note, social services were consulted and excluded child abuse. Due to the severity of his injury, plastic surgery was consulted. The physical examination, with tongue at rest inside the mouth, revealed a 3.5 cm midline full-thickness laceration that extended to between one-half to two-thirds of the free tongue. No foreign bodies were noted, and dentition was intact (Fig. 1).

We recommended emergent operative repair under general anesthesia. Signed informed consent was obtained from the patient's mother. The operation was performed by the senior author with 15 years of Attending Plastic Surgery experience. Once anesthetized with an endotracheal tube, the tongue was exposed revealing a longitudinal full-thickness laceration of the tongue through the median fibrous septum. Two 0- Silk sutures were used to align the two halves of the tongue; devitalized edges were conservatively debrided, and hemostasis was obtained. Buried interrupted 2-0 Vicryl sutures were used to approximate the middle muscular layer and 3-0 chromic running sutures were used along dorsal and ventral epithelium.

**Fig. 2:**
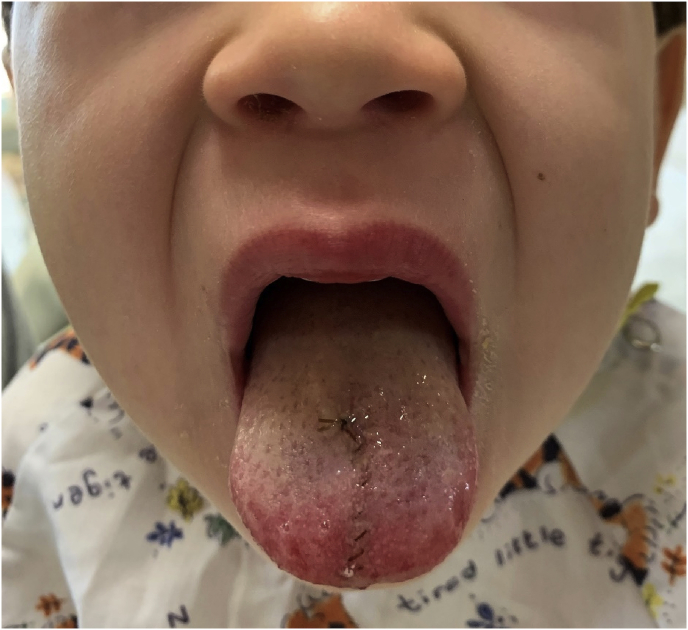
Postoperative Day #1. Status post repair of a complex tongue laceration.

**Fig. 3:**
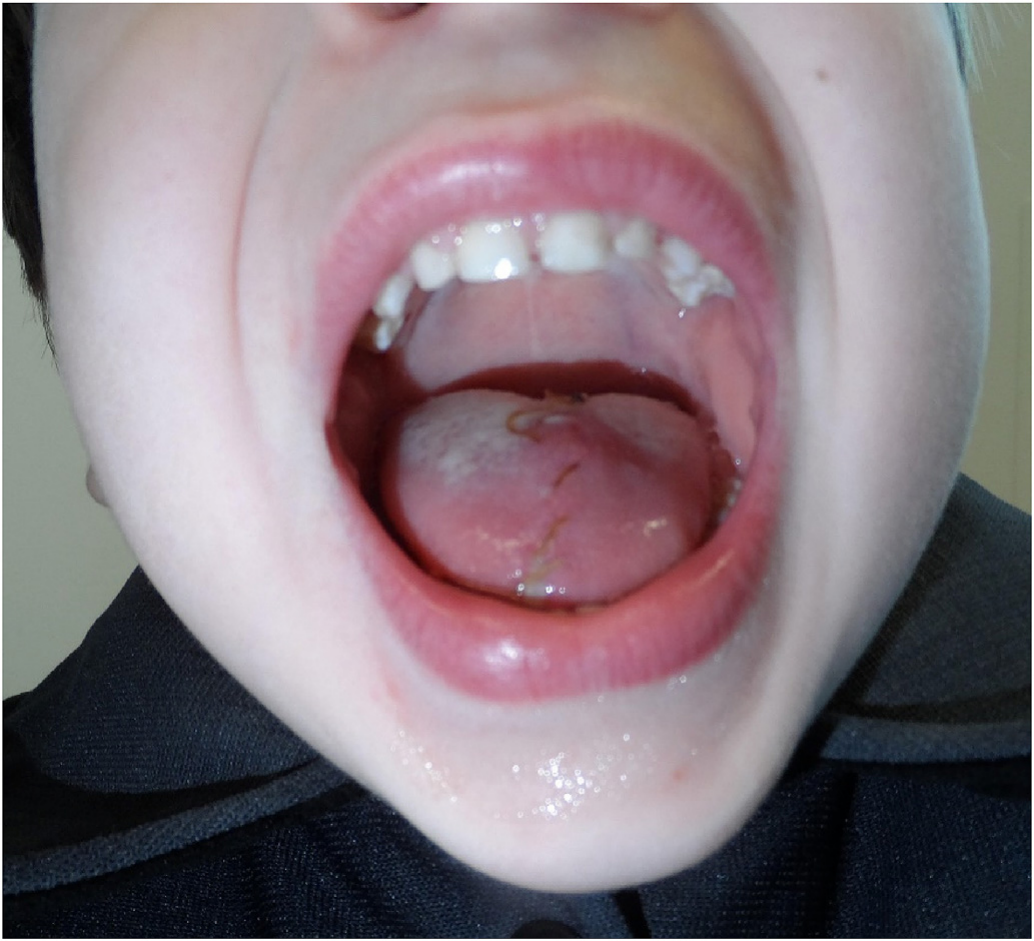
2-Week follow-up. Status post repair of a complex tongue laceration.

The patient was monitored overnight for airway concerns and discharged the next morning (Fig. 2). Early tongue mobilization was encouraged, non-steroidal analgesia was used, and a liquid diet was started. At the 2-week follow up visit, his healing was uneventful without any distortion (Fig. 3). At a 3-month follow-up visit, parents reported that he was “back to normal” and denied any loss of sensation, taste, mobility, or speech.

## 3 Discussion

The severity of tongue injuries ranges from minor lacerations to complete amputation. The short-term sequela may include pain, inability to speak, bleeding [1], disfigurement, loss of function, infection, swelling, and airway compromise [2,4]. Significant trauma at the tongue base may injure the Hypoglossal nerve affecting long-term function [2] including impaired speech [3]. Healing with secondary intention may lead to fibrosis and long-term distortion [2]. While there is consensus in the management of extreme presentations, there is still debate whether less severe lacerations should be repaired with sutures or allowed to heal with secondary intention [3,7].

Some authors have advocated nonoperative healing by secondary intention for lateral and tip location [2] and non-gaping wounds [4]. Even though a mucosal application is not indicated, tissue adhesives such as 2-octyl cyanoacrylate have been reported as treatment for tongue trauma [3].

A prospective study of 28 pediatric patients with tongue trauma found the typical laceration was self-limiting and did not require intervention [4]. A retrospective study of 73 pediatric tongue trauma patients was used to develop Zurich Tongue Scheme to guide clinicians in determining when suturing is necessary. No complex or bisecting injuries were noted. The tongue border was involved in 69.9% of their cases and injury was full-thickness in 31.5% of the cases. The authors recommend suturing for large flaps, uncontrolled bleeding, lacerations greater than 2 cm, or involvement of tongue tip [3].

In general, interventions for severe tongue lacerations should begin as soon as possible, preferably within 8 hours of injury as delay beyond 24 hours can worsen the outcome [2,4,8]. The technical aspects of the operation largely determine the postoperative functional and cosmetic recovery. The suture technique must close the muscular planes with absorbable sutures to stop the hemorrhage and prevent hematoma formation [1] with buried absorbable sutures to relieve tension [2]. Post-repair recommendation is a liquid diet with rapid progression, oral hygiene, and early mobility [2]. The potential complications of suturing include scarring, suture granuloma, and lisping [3].

In most trauma cases, the tongue is caught in between teeth and results in a horizontal- oriented injury [4]. The injury in the present case is unique in being longitudinal and midline. The parents noted their basement is cemented with expansion joints separating concrete slabs. They suspect the child fell in a way that his tongue got caught between the teeth and expansion joint to cause the bifid pattern.

An acquired bifid tongue in a child is an unusual presentation. Most cases of the pediatric bifid tongue are congenital resulting from orofacial digital syndromes type I, II, IV, and IV or syndromic cases such as Opitz G BBB syndrome, Klippel-Fiel anomaly or Larsen Syndrome [9]. This congenital anomaly may also be seen in infants of Diabetic Mother Syndrome [10].

An acquired bifid tongue is more common in adults arising as a complication of tongue piercing or less frequently from intentional splitting. Both are forms of body art modifications that can lead to complications requiring surgical repair [11].

Given the severity of the injury in our patient, surgical repair under anesthesia was the obvious choice. In the operating room, the devitalized edges were freshened, and hemostasis was obtained. The tongue “halves" were orientated. Instead of selectively identifying individual muscle bundles, a single layer of buried absorbable sutures was used followed by a second layer of running absorbable sutures. We believe, the midline location protected the neurovascular supply and resulted in a favorable outcome.

## 4 What does this case report add?

A unique presentation and repair of an acquired pediatric bifid tongue secondary to trauma. To the best of our knowledge, this is the first such case to be reported in the English literature.

## 5 Conclusion

The presentation of tongue trauma varies, from minor injury requiring healing with secondary intention to a major deformity requiring surgical repair. In our pediatric patient, a midline full-thickness laceration through the median fibrous septum resulted in an acquired bifid tongue. The patient underwent immediate surgical repair. Given the midline location, neurovascular supply was protected, and the patient enjoyed essentially a complete, uneventful recovery.

## Ethical approval

No Institutional Review Board approval needed for case report at our institution.

## Sources of funding

No sponsor and no funding

## Author contribution

AK, senior author, performed the intervention. Both authors, AK and KK, contributed to manuscript. Both authors have read and agreed with the manuscript.

## Registration of research studies

1. Name of the registry: NA. This is a case report of one patient.
2. Unique Identifying number or registration ID:
3. Hyperlink to your specific registration (must be publicly accessible and will be checked):

## Guarantor

Ajaipal s. Kang, MD FACS.

## Consent

Written informed consent was obtained from the patient for publication of this case report and accompanying images. A copy of the written consent is available for review by the Editor-in-Chief of this journal on request.

Written consent is from child's parent.

## Financial disclosure

None.

## Patient/guardian consent

The child's parents have given consent for possible publication of this case report.

## Provenance and peer review

Not commissioned, externally peer reviewed.

## Declaration of competing interest

No conflict of interest from both authors.
